# Calcineurin Controls Expression of EAAT1/GLAST in Mouse and Human Cultured Astrocytes through Dynamic Regulation of Protein Synthesis and Degradation

**DOI:** 10.3390/ijms21062213

**Published:** 2020-03-23

**Authors:** Giulia Dematteis, Elena Restelli, Roberto Chiesa, Eleonora Aronica, Armando A Genazzani, Dmitry Lim, Laura Tapella

**Affiliations:** 1 Department of Pharmaceutical Sciences, Università del Piemonte Orientale "Amedeo Avogadro", 28100 Novara, Italy; 2 Department of Neuroscience, Istituto di Ricerche Farmacologiche Mario Negri IRCCS, 20156 Milano, Italy; 3 Amsterdam UMC, University of Amsterdam, Department of (Neuro) Pathology, 1105 AZ Amsterdam, The Netherlands; 4 Stichting Epilepsie Instellingen Nederland (SEIN), 2103 Heemstede, The Netherlands

**Keywords:** astrocytes, calcineurin, GLAST, protein synthesis, protein degradation, proteostasis

## Abstract

Alterations in the expression of glutamate/aspartate transporter (GLAST) have been associated with several neuropathological conditions including Alzheimer’s disease and epilepsy. However, the mechanisms by which GLAST expression is altered are poorly understood. Here we used a combination of pharmacological and genetic approaches coupled with quantitative PCR and Western blot to investigate the mechanism of the regulation of GLAST expression by a Ca^2+^/calmodulin-activated phosphatase calcineurin (CaN). We show that treatment of cultured hippocampal mouse and fetal human astrocytes with a CaN inhibitor FK506 resulted in a dynamic modulation of GLAST protein expression, being downregulated after 24–48 h, but upregulated after 7 days of continuous FK506 (200 nM) treatment. Protein synthesis, as assessed by puromycin incorporation in neo-synthesized polypeptides, was inhibited already after 1 h of FK506 treatment, while the use of a proteasome inhibitor MG132 (1 μM) shows that GLAST protein degradation was only suppressed after 7 days of FK506 treatment. In astrocytes with constitutive genetic ablation of CaN both protein synthesis and degradation were significantly inhibited. Taken together, our data suggest that, in cultured astrocytes, CaN controls GLAST expression at a posttranscriptional level through regulation of GLAST protein synthesis and degradation.

## 1. Introduction

Glutamate is a principal excitatory neurotransmitter in the central nervous system (CNS) and its correct homeostasis, which includes uptake and release, is of paramount importance for correct function of the CNS. Released by glutamatergic terminals at the excitatory synapses, glutamate acts on both ionotropic and metabotropic receptors to transmit signals and induce plasticity [1]. Uptake of glutamate is mediated by a family of five excitatory amino acid transporters (EAAT1-5) [1] differentially expressed in different brain regions and cell types, all acting with the same stoichiometry: Importing one glutamate molecule by the co-transport of three sodium ions and one proton, while exporting one potassium ion [2]. Astrocytes, being the principal glutamate-metabolizing cells in the CNS, are endowed by two high affinity glutamate transporters, glutamate/aspartate transporter EAAT1/GLAST and glial high affinity glutamate transporter 1 EAAT2/Glt-1, which are preferentially expressed at the membrane surface [3]. In healthy adult hippocampus EAAT2/Glt-1 is preferentially expressed, while in cultured hippocampal astrocytes EAAT1/GLAST becomes predominant [4,5,6]. 

Alterations of Glt-1 and GLAST expression and activity have been associated with many neurological disorders such as schizophrenia, epilepsy and Alzheimer’s disease (AD) [7,8]. Therefore, understanding of how the EAATs expression is regulated is important for identification of therapeutic targets to treat pathologies correlated with alterations of glutamate transporters. 

Expression of Glt-1 and GLAST has been shown to be regulated at both transcriptional and translational levels depending on experimental and/or pathological context [9,10]. At the transcriptional level Gtl-1 has been shown to be inversely regulated by calcineurin (CaN)—nuclear factor of activated T-cells (NFAT) axis [11,12]. Whether GLAST is also regulated by Ca^2+^ and CaN is currently not known.

CaN, a Ca^2+^/calmodulin-activated phosphatase, is a heterodimer, composed of a catalytic subunit CaNA and an obligatory regulatory subunit CaNB. Cooperative binding of Ca^2+^ to CaNB and of Ca^2+^/calmodulin complex to CaNA leads to displacement of the auto-inhibitory domain permitting the interaction of CaN with its substrates. CaN activates gene transcription through direct activation of NFAT [13] and modulation of the activation cascade of NF-kB [14]. CaN is also able to regulate activity, interaction, or location of target proteins directly through dephosphorylation [15]. 

Recently we have reported that in cultured hippocampal astrocytes from an astroglial CaN knock-out mouse, GLAST protein was upregulated while mRNA levels were not changed [6]. Thus, we thought to investigate the mechanism of CaN-dependent up-regulation of GLAST in cultured astrocytes. Our results suggest that CaN in astrocytes regulates expression of GLAST at the posttranscriptional level through dynamic regulation of protein synthesis and degradation.

## 2. Results

### 2.1. Pharmacological CaN Inhibition Results in a Dynamic Modulation of GLAST Protein in Both Mouse and Human Astrocytes 

**Figure 1:**
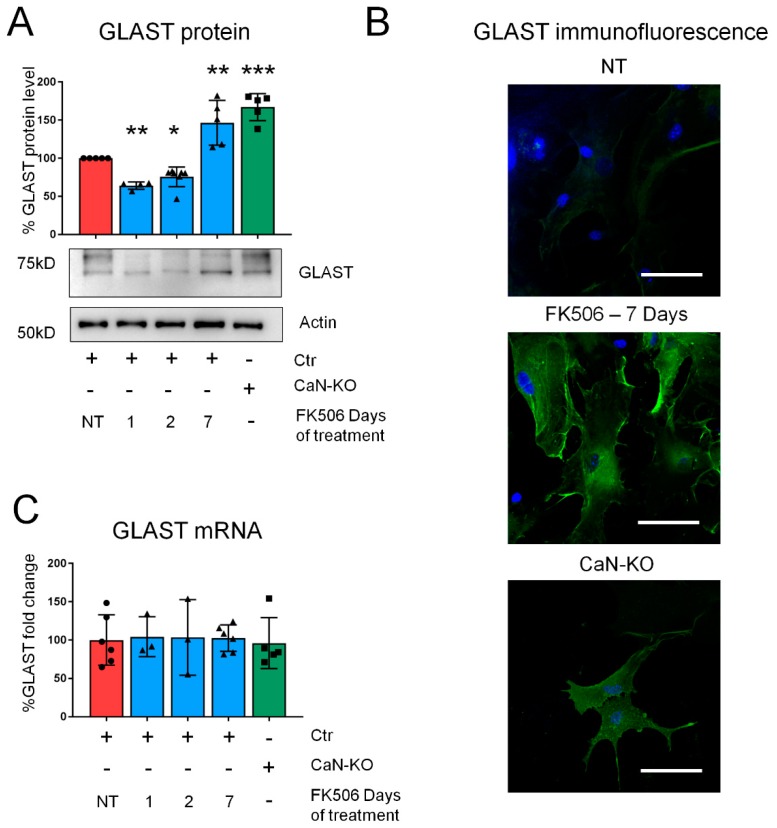
CaN inhibition dynamically regulates GLAST expression in mouse hippocampal astrocytes. (**A**) Glutamate/aspartate transporter (GLAST) protein expression in primary cultures of hippocampal astrocytes from control not-treated (Ctr, NT) Ctr astrocytes treated with FK506 200nM for 1, 2 and 7 days and of cultured astrocytes from mice with an astrocyte-specific deletion of Ca^2+^/calmodulin-activated phosphatase calcineurin (CaN-KO). Data are expressed as mean ± SD, 5 independent cultures were used for each condition. * *p* < 0.05; ** *p* < 0.01; *** *p* < 0.001, one way ANOVA with Tukey post hoc test. (**B**) Immunofluorescence anti-GLAST images of Ctr-NT, Ctr treated with FK506 200 nM for 7 days and CaN-KO. Green, GLAST; blue, DAPI (4′,6-diamidino-2-phenylindole). Bar, 50 μm. (**C**) Real-time PCR of GLAST from primary astrocytes from Ctr-NT, Ctr treated with FK506 200 nM for 1, 2, and 7 days, and from astrocyte-specific CaN-KO mice. Values represent mean  ±  SD ΔC(t) of gene/S18 of 6 independent cultures for each condition.

Starting from the observation of increased GLAST expression in cultured hippocampal astrocytes with a genetic deletion of CaN (CaN-KO) [6] we investigated if pharmacological inhibition of CaN with the specific and clinically relevant inhibitor FK506 (200 nM) would result in GLAST overexpression. Surprisingly, after 1 or 2 days of treatment GLAST was, instead, downregulated (Figure 1A). We have reasoned that in CaN-KO astrocytes, the upregulation of GLAST would represent an end-point of the effect of CaN deletion. Therefore, we followed GLAST expression until 7 days of FK506 treatment and found that at 7 day GLAST was significantly up-regulated resembling the upregulation observed in CaN-KO astrocytes (Figure 1A). In an immunocytochemical analysis the downregulation of GLAST at 1 or 2 days of FK506 treatment was not evident (not shown) likely due to the low basal level of GLAST expression, while at the 7th day of treatment GLAST fluorescence was strongly increased (Figure 1B). Next we investigated if the biphasic dynamics of GLAST expression upon CaN inhibition was due to changes in mRNA levels, e.g., if CaN regulated GLAST expression at transcriptional level. Quantitative PCR results show that the mRNA levels of GLAST were not different at any time point of FK506 treatment (Figure 1C), corroborating the absence of transcriptional regulation in CaN-KO astrocytes [6]. 

**Figure 2:**
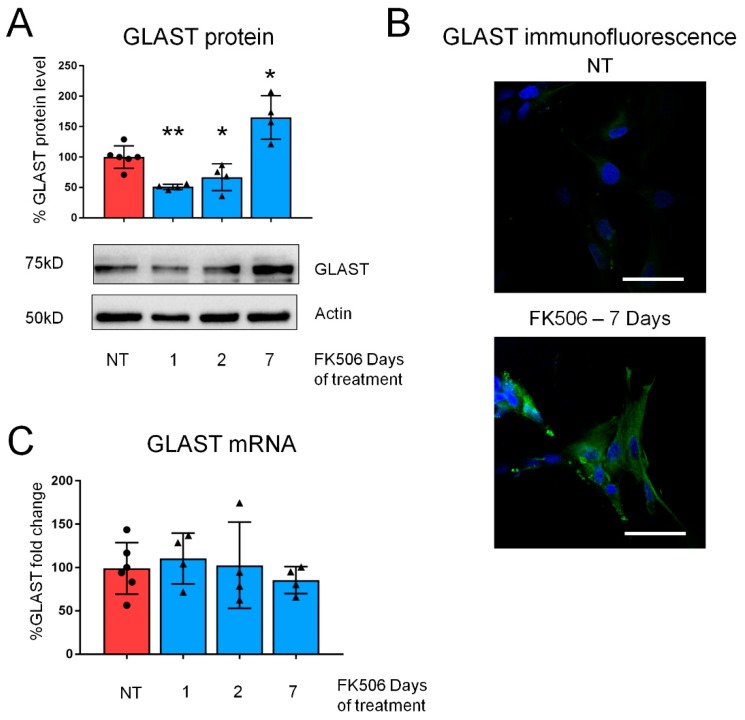
CaN inhibition dynamically regulates GLAST expression human astrocytes. (**A**) GLAST protein expression in primary cultures of human astrocytes untreated and treated with FK506 (200 nM) for 1, 2, and 7 days. Data are expressed as mean ± SD. * *p* < 0.05; ** *p* < 0.01; one way ANOVA with Tukey post hoc test. (**B**) Immunofluorescence images of human primary astrocytes, untreated and treated with FK506 for 7 days, stained with anti-GLAST antibody (green). Nuclei are stained with DAPI (blue). Bar, 50 μm. (**C**) Real-time PCR of GLAST from human primary astrocytes, untreated and treated with FK506 (200 nM) for 1, 2 and 7 days. Values represent mean  ±  SD ΔC(t) of gene/S18 of 4 independent experiments for each condition.

To consolidate this finding and to demonstrate that the effect of CaN inhibition was not limited exclusively to mouse astrocytes, we treated with FK506 (200 nM) fetal human cultured astrocytes. The observed dynamics of GLAST expression during 7 days treatment was very similar to that of mouse hippocampal astrocytes showing significant downregulation at 1 st and 2 nd days and upregulation after 7 days of treatment (Figure 2A). Similarly to mouse astrocytes, immunofluorescent analysis revealed strong increase in immunoreactivity to GLAST at 7 days (Figure 2B), while mRNA levels did not change at any time-point (Figure 2C).

### 2.2. CaN Modulates GLAST Protein Expression through the Regulation of the Equilibrium between Protein Synthesis and Degradation

**Figure 3:**
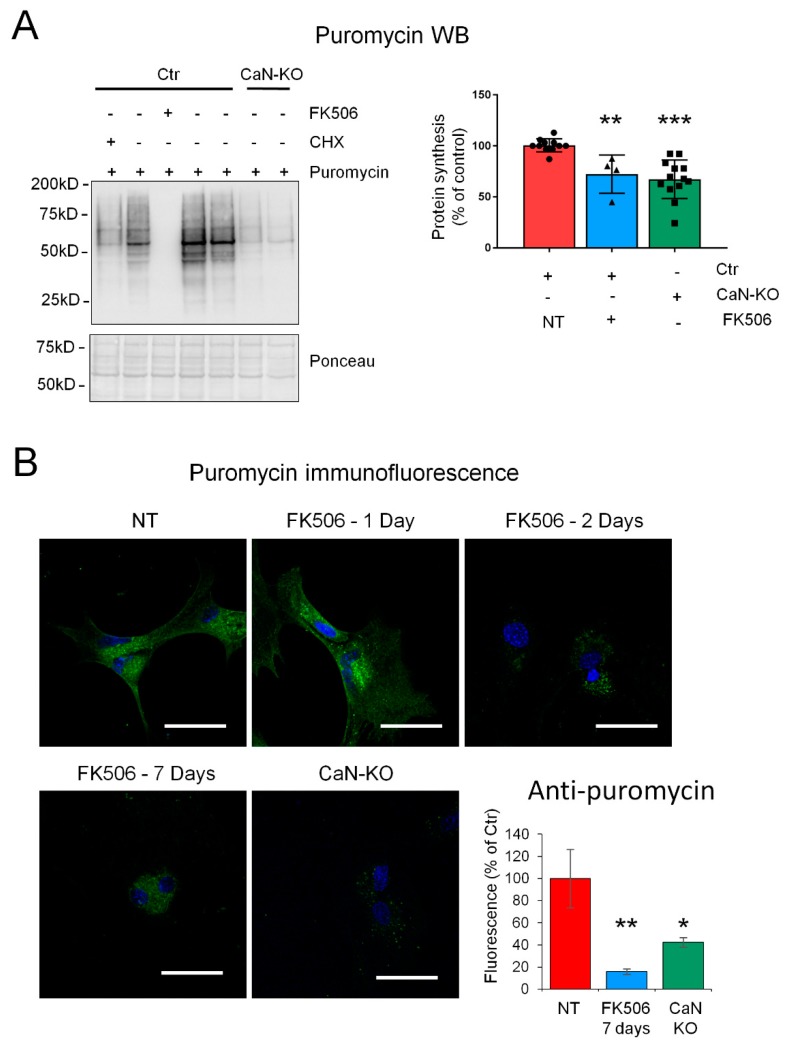
CaN inhibition permanently suppresses protein synthesis in cultured astrocytes. (**A**) Ctr and CaN-KO primary astrocytes were pulsed with puromycin for 3 h. Ctr were also treated with FK506 for 1 h to inhibit CaN activity. Where indicated, cycloheximide (CHX, 10 μM) was added ten minutes before adding puromycin. Anti-puromycin antibody was used to detect neo-synthesized peptides. Ponceau staining was used for band normalization. Histogram shows quantification of anti-puromycin detected bands. Data are expressed as mean  ±  SD, 5, 4, and 11 independent cultures were used for Ctr, FK506 treated and CaN-KO samples, respectively. ** *p* < 0.01; *** *p* < 0.001, one way ANOVA with Tukey post hoc test. (**B**) Immunofluorescence images of Ctr, Ctr treated with FK506 (200 nM) for 1, 2 and 7 days and CaN-KO, stained with anti-puromycin antibody (green). Nuclei are stained with DAPI (blue). Bar, 25 μm. Data are expressed as mean  ±  SD; * *p* < 0.05; ** *p* < 0.01, one way ANOVA with Tukey post hoc test.

Given the absence of alterations in GLAST mRNA levels we reasoned that the dynamic modulation of GLAST protein may be a result of alterations of protein synthesis rate. To investigate this, we employed a recently developed method of surface sensing of translation (SUnSET) which is based on puromycin incorporation in neo-synthetized peptides [16] with consequent detection by anti-puromycin immunoreactivity either by immunoblot or immunofluorescence. We found that incorporation of puromycin was drastically inhibited already 1 h after addition of FK506 (Figure 3A). Longer FK506 incubation somewhat resulted in the appearance of a smear in anti-puromycin probed membranes which rendered quantification of the band intensity unreliable. Nevertheless, we found that incorporation of puromycin in CaN-KO astrocytes was also significantly inhibited (Figure 3A), suggesting that the impairment of protein synthesis in FK506-treated astrocytes was a result of inhibition of CaN activity. To follow the long-term effect of pharmacological CaN inhibition, we took advantage of immunofluorescent puromycin labelling. Interestingly, beginning from the second day of FK506 treatment, a drastic reduction of puromycin immunoreactivity was observed in all time points, including 7 days-time-point, suggesting an irreversible inhibition of proteins synthesis upon blockade of CaN activity (Figure 3B). In line with this, fluorescence intensity of anti-puromycin staining in CaN-KO astrocytes was also significantly lower compare to control/not-treated astrocytes. Thus, the downregulation of GLAST protein during the first days of FK506 treatment may be a result of the inhibited protein synthesis downstream of pharmacological inactivation of CaN in astrocytes. However, the upregulation of GLAST after long-term FK506 treatment or in astrocytes with KO of CaN was at odds with the inhibition of protein synthesis.

**Figure 4:**
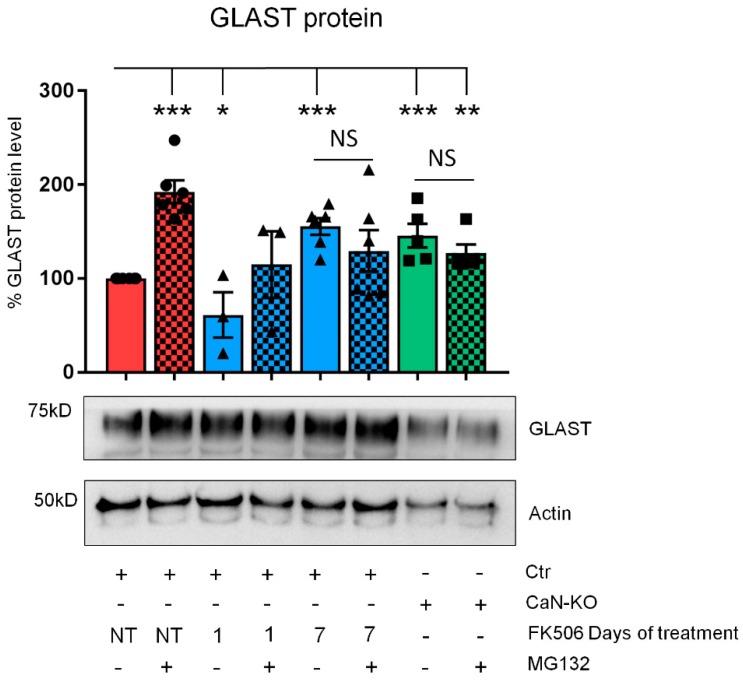
Long-term, but not short-term CaN inhibition suppresses GLAST protein degradation. GLAST protein expression in primary cultures of hippocampal astrocytes from Ctr, Ctr treated with FK506 (200 nM) for 1 and 7 days and from astrocytes-specific CaN-KO mice. Where indicated, MG132 was added 3 h before lysis. Data are expressed as mean  ±  SD, 6 independent cultures were used for both Ctr and Ctr treated with FK506 for 7 days, 5 for CaN-KO, and 3 for Ctr treated with FK506 for 1 day. * *p* < 0.01; ** *p* < 0.01; *** *p* < 0.001, unpaired two-tailed Student’s t-test.

Because of the amount of a protein in the cell, at a given transcriptional rate, is determined by the equilibrium of protein synthesis and protein degradation [17], we hypothesized that the augmented GLAST protein in concomitance with the inhibition of protein synthesis may result from the impairment of protein degradation system, namely proteasome [18]. To test this hypothesis, we treated astroglial primary cultures with MG132 (1 μM, 3 h), a potent proteasome inhibitor [19]. Expectedly, treatment of control astrocytes with MG132 induced a significant increase in GLAST protein expression (Figure 4) as it did also after 1 day of FK506 treatment, indicating that proteasome was active in these conditions. However, neither in astrocytes at 7 th day of FK506 treatment nor in CaN-KO astrocytes, increase of GLAST expression was observed (Figure 4), suggesting that both long-term FK506 treatment and genetic CaN ablation resulted in inhibition of proteasome activity.

### 2.3. Upregulation of GLAST Protein in Astroglial CaN-KO Hippocampal Synaptosomes

**Figure 5:**
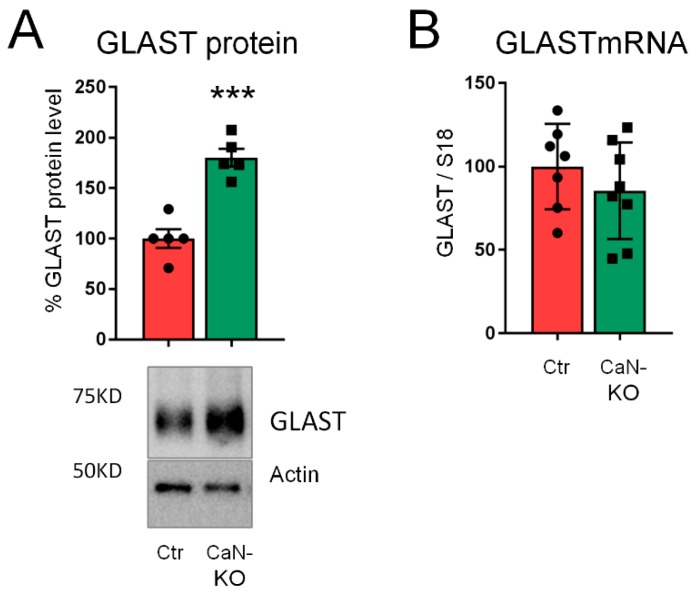
GLAST protein, but not mRNA, is upregulated in hippocampal synaptosomes from astroglial CaN-KO mouse. (**A**) GLAST protein expression in hippocampal synaptosomes from control (Ctr) and astroglial CaN-KO mice (CaN-KO). Data are expressed as mean  ±  SD, 5 independent synaptosomal preparations, each of which was a pool from two animals, were used for both Ctr and CaN-KO. *** *p* < 0.001, unpaired two-tailed Student’s t-test. (**B**) Real-time PCR of GLAST from hippocampi of Ctr and astrocytic CaN-KO mice. Values represent mean  ±  SD ΔC(t) of gene/S18 of 7 independent samples for each genotype.

To consolidate our findings and to investigate if in vivo deletion of CaN may result in GLAST upregulation, we used WB to quantify GLAST protein in hippocampal synaptosomal fractions in which GLAST is enriched in perisynaptic astrocytic processes at tripartite synapse [20,21]. According to our in vitro data, GLAST was significantly up-regulated in synaptosomes from astroglial CaN-KO mouse compare with control preparations (Figure 5A), while no changes of GLAST mRNA levels were detected (Figure 5B).

Altogether, our data suggest that inhibition of CaN in astrocytes results in deregulation of both protein synthesis and degradation, albeit with different time-scale, resulting in dynamic modulation of GLAST protein expression. 

## 3. Discussion

**Figure 6:**
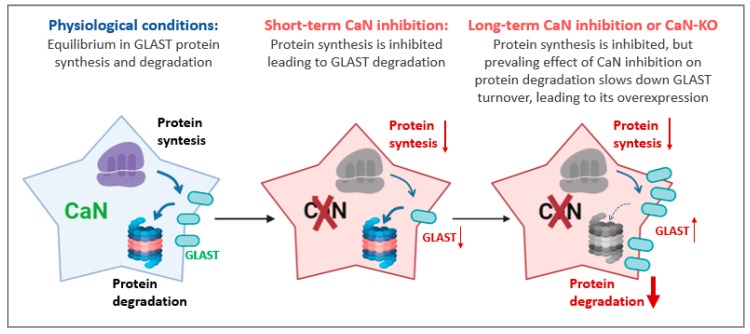
Scheme of the proposed mechanism for the time-dependent dynamic modulation of GLAST protein expression upon inhibition of CaN in astrocytes. Left astrocyte, protein synthesis and protein degradation are in equilibrium maintaining constant expression of GLAST. Middle astrocyte, short-term CaN inhibition by FK506 results in a prevalent inhibition of protein synthesis resulting in downregulation of GLAST. Right astrocyte, long-term pharmacological CaN inhibition or knock-out of CaN in astrocytes result in prevailing effect on the inhibition of protein degradation over the inhibition of protein synthesis, resulting in slowing down the GLAST turnover and its accumulation. The time-dependent effect of CaN inhibition on dynamics of other proteins in the cell may differ from that of GLAST.

The present work was designed to investigate the mechanism(s) of upregulation of glutamate/aspartate transporter GLAST in cultured hippocampal astrocytes [6]. We found that, while astrocyte-specific genetic ablation of CaN leads to a constitutive increase of GLAST protein, its pharmacological inhibition produces a biphasic modulation showing reduction at 1–2 days but upregulation of GLAST protein expression at 7th day after beginning of treatment. Such a modulation occurred at a posttranscriptional level through differential inhibition of protein synthesis and degradation (Figure 6).

Uptake of glutamate, released during neuronal activity and synaptic transmission, is an important aspect of astroglial patho-physiology [3] and the fine tuning of this process by regulation of expression of glutamate transporters contributes to glutamate homeostasis, while deregulation of glutamate transporters expression in many pathological conditions has been documented [9,10]. Expression of GLAST has been shown to be modulated by several physiologically and pathologically relevant agents. Thus, growth factors and steroid hormones upregulate GLAST in cultured astrocytes, at both mRNA and protein levels [22,23]. Instead, phorbol esthers and thrombin downregulate GLAST in cultured astrocytes [24,25,26]. In neuron-astrocyte co-cultures, GLAST expression has been shown to be dynamically modulated by neuronal activity and depended on the state of neuronal maturation [27]. Such a modulation has been proposed to depend on astrocyte’s interaction with substrate and other cells [28], while whether this requires de-novo GLAST mRNA synthesis is not known. However, it has been demonstrated that transcriptional upregulation of GLAST in response to amitriptyline requires activation of transcription factor NF-kB [29] and that GLAST promoter contains NF-kB binding sites [30]. We and others have shown that, in astrocytes, cytokine- and Aβ-induced activation of NF-kB requires activation of CaN [31,32,33], although, whether CaN activation of NF-kB is required for regulation of GLAST expression is not clear. Here we show that both in vitro and in vivo blockade of CaN activity by pharmacological and genetic means leads to modulation of GLAST expression without changes of its mRNA levels, suggesting a posttranscriptional mechanism. However, CaN inhibition may not necessarily affect same pathways induced by CaN over-activation. Thus, pathology-related over-activation of CaN in astrocytes has been repeatedly associated with downstream transcriptional remodeling [12,31,34]. Therefore, it could be speculated that transcriptional regulation of GLAST may be activated in conditions of experimental and/or pathological over-activation of CaN in astrocytes, while in resting/physiological conditions the regulation occurs through CaN modulation of protein synthesis and degradation, although experimental proof of this speculation is a matter of future work. 

The central finding of this work is that both genetic ablation and pharmacological inhibition of CaN drastically inhibited both protein synthesis and degradation in cultured astrocytes. Here we investigate this focusing on GLAST expression. However, there may be more general interpretation of this finding. The expression dynamics of other than GLAST proteins may not necessarily be modulated as those of GLAST. This may depend on the posttranslational modifications and on the protein turnover rate [35]. Therefore, it may be suggested that the inhibition of CaN astrocytes will likely result in dysproteostasis [17] rather than massive decrease/increase in protein mass, i.e., some proteins will be upregulated while some downregulated albeit with different time-scale dynamics, yet other proteins will not change their expression levels. This may be reflected by the fact that at a given total proteins concentration in our samples, actin expression did not change at any condition and time-point.

Taken separately, involvement of CaN in protein synthesis and degradation processes is not novel. Thus, direct CaN modulation of protein synthesis has been reported previously and, at the level of translational machinery, at least four factors, eukaryotic translation initiation factor 2B (eIF2B), eukaryotic translation initiation factor 4E binding protein 1 (4EBP1), eukaryotic translation initiation factor 4F (eIF4F) and eukaryotic translation elongation factor 2 (eEF2), have been suggested to be directly regulated by CaN [36]. Protein degradation has also been associated with CaN activity through its involvement in lysosomal activity [37], autophagy [38,39], ER stress, and unfolded protein response (UPR) [40,41]. Association of CaN activation which ubiquitin-ligase/proteasome system has also been suggested [42,43]. In astrocytes, CaN has been shown to regulate UPR through direct interaction with protein kinase RNA-like endoplasmic reticulum kinase (PERK) [44,45]. Recently, activation of PERK-mediated UPR in astrocytes has been shown to induce neuronal degeneration [46]. As it was mentioned above, astrocytes are the main homeostatic cells in the CNS, which use the cell-cell interaction-mediated Ca^2+^ signaling to finely tune their homeostatic activities [47]. In light of this, and of the discussed above pathways which may be affected by the over-activation of CaN, it can be speculated that, in neurodegenerative conditions, the astroglial CaN over-activation may result in a pattern of protein expression, at least in part, similar to that produced by the inhibition of CaN in astrocytes, albeit, this may differ substantially between brain cell types. Such an effect may be brought by the concomitance of the CaN activation with other known and unknown mechanisms, affecting the proteostasis machinery, which are activated by the neurodegenerative process. One could suggest that the neuropathological outcome of the disease will not depend on the action of a single molecule/enzyme (e.g., over-activated CaN), but on the result of such an intricate interaction with parallel/downstream mechanisms bringing the proteostasis machinery to collapse.

In conclusion, our results suggest that astroglial CaN, through modulation of a dynamic equilibrium between protein synthesis and degradation, may regulate proteostasis of astrocytes and thereby, their homeostatic activities including uptake of glutamate, in which GLAST is primarily involved. This provides a framework for investigation of the role of astroglial CaN in regulation of the CNS proteostasis.

## 4. Materials and Methods 

### 4.1. Astrocyte-Specific CaN KO Mice

Generation and handling of conditional CaN knockout (KO) in GFAP-expressing astrocytes has been described previously [6]. Mice were housed in the animal facility of the Università del Piemonte Orientale, with unlimited access to water and food. Animals were managed in accordance with European directive 2010/63/UE and with Italian law D.l. 26/2014. The procedures were approved (3 June 2016) by the local animal-health and ethical committee (Università del Piemonte Orientale) and were authorized by the national authority (Istituto Superiore di Sanità; authorization numbers N. 77-2017 and N. 214-2019). All efforts were made to reduce the number of animals by following the 3R’s rule.

### 4.2. Mouse Astrocytic Primary Cultures 

Primary astroglial cultures were obtained by extracting hippocampi from either ACN-Ctr or KO mouse pups at postnatal day 1-5 (P1-P5). Hippocampi were dissected, in cold HBSS, from pups brains and dissociated by incubation with trypsin (0.5 mg/mL, 37 °C, 20 min) followed be a gentle triturating and resuspension in Dulbecco’s Modified Eagle’s Medium (DMEM, Sigma, Milan, Italy)—high glucose, supplemented with 10% foetal bovine serum (FBS), 2 mg/mL glutamine, 10 U/mL penicillin and 100 mg/mL streptomycin (Sigma, Milan, Italy). Each pup was processed separately and plated pre-treated with 0.1 mg/mL poly-L-lysine (PLL, Sigma, Milan, Italy). At sub-confluence (5–10 days in vitro), cells were detached with trypsin and pleated for experiments. 

### 4.3. Fetal Human Primary Astrocytes

Primary fetal astrocyte-enriched cell cultures were derived from human fetal brain tissue (14–20 weeks of gestation) obtained from medically induced abortions. All material was collected from donors from whom a written informed consent for the use of the material for research purposes. Tissue was obtained in accordance with the Declaration of Helsinki and the Amsterdam UMC Research Code provided by the Medical Ethics Committee. Tissue samples were collected in astrocyte medium: DMEM/HAM F10 (1:1; Gibco/ThermoFisher Scientific, Waltham, MA, USA), supplemented with 100 units/mL penicillin, 100 μg/mL streptomycin and 10% fetal calf serum (Gibco, Life Technologies, Grand Island, NY, USA). Cell isolation was performed as previously described [48].

### 4.4. Pharmacological Treatments

Cultured astrocytes were plated in a 6 well plate pre-treated with 0.1 mg/mL poly-L-lysine (PLL, Sigma, Milan, Italy), and grown until 60% of confluence. 200 nM FK506 (TOCRIS, Cat. 3631) was added 24 h, 48 h or 1 week prior to lysis. In the 1 week treatment setting FK506 was re-added at 3rd day of treatment.

For assessment of proteasome activity, 3 h before lysis, cells were treated with 1 µM (R)-MG132 (Cat. 6033, TOCRIS, Britol, UK). 

### 4.5. Puromycin Incorporation Method (Surface Sensing of Translation, SUnSET)

Cultured astrocytes were incubated with 4 µM puromycin dihydrochloride (Cat. P8833, Sigma, Milan, Italy) supplemented in normal medium at 37 °C with 5% CO_2_ for 3 h [16]. Subsequently, cell’s lysates were subjected to Western blot assay or fixed for immuno-histochemistry.

### 4.6. Immunofluorescence and Confocal Microscopy

Primary mouse hippocampal and foetal human astrocytes, grown on 13 mm glass coverslips and treated as indicated, were fixed in 4% formaldehyde and 4% sucrose, permeabilized (7 min in 0.1% Triton X-100 in phosphate-buffered saline (PBS)), blocked in 1% gelatin, and immunoprobed with an appropriate primary antibody (diluted in PBS supplemented with 1% gelatine) over night at 4 °C. After 3 times washing in PBS, an Alexa-conjugated secondary antibody (1:300 in PBS supplemented with 1% gelatine) was applied for 1 h at room temperature (RT). The following primary antibodies were used: anti-GLAST (rabbit, 1:100, Cat. NB100-1869, Novusbio, Abingdon, UK), anti-puromycin (1:200, Millipore, Cat. MABE343). Secondary antibodies were as follows: Alexa Fluor 488 anti-mouse IgG, Alexa Fluor 555 anti-rabbit IgG (all secondary antibodies were from Molecular Probes, Life Technologies, Monza, Italy). Nuclei were counter-stained with 4′,6-diamidino-2-phenylindole (DAPI). Images were acquired using an FV-500 Olympus (Tokyo, Japan) laser confocal scanning system with a 60X oil immersion objective. Immunofluorescence signal intensity per cell was measured with NIH ImageJ software v1.52p and was calculated as corrected total cell fluorescence (CTCF) = Integrated Density—(Area of selected cell X Mean fluorescence of background readings).

### 4.7. Preparation of Synaptosomes

Synaptosomal fractions were isolated by differential centrifugation using standard protocols [49]. Briefly, mice were anesthetized and sacrificed by decapitation. Hippocampi were rapidly dissected and placed into ice-cold homogenization buffer containing 50 mM MOPS, pH 7.4, 320 mM sucrose, 0.2 mM DTT, 100 mM KCl, 0.5 mM MgCl2, 0.01 mM EDTA, and 1 mM EGTA, protease inhibitor cocktails (PIC, Calbiochem, San Diego, CA, USA) and phosphatase inhibitor Na_3_VO_4_ 1 µM. All subsequent steps were performed at 4 °C. The hippocampus were microdissected and homogenized in 1:10 *w/v* homogenization buffer with 12 strokes in a Teflon-glass douncer. The homogenates centrifuged for 10 min at 800× *g* followed by centrifugation of the supernatant at 9200× *g* for 15 min. The resulting P2 pellet, representing the crude synaptosomal fraction, was solubilized in lysis buffer. 

### 4.8. Cell lysis and Western blot

Astroglial cultures were lysed with 100 µL of lysis buffer (50 mM TrisHCl (pH 67.4), sodium dodecyl sulphate (SDS) 0.5%, 5mM EDTA), 10 µL of protease inhibitors cocktail (PIC, Calbiochem) and 1 µL of phosphatase inhibitor Na3VO4 1 M; removed with a scraper, and collected in a 1.5 mL tube. Lysates were then boiled for 5’, and quantified with QuantiPro BCA Assay Kit (cat. SLBF3463, Sigma, Milan, Italy).

30 µg of proteins were mixed with the right amount of Laemmli Sample Buffer 4X (Bio-Rad, Segrate, Italy), and boiled for 5’. Then samples were loaded on a 12% polyacrylamide-sodium dodecyl sulphate gel for electrophoresis. Proteins were transferred onto nitrocellulose membrane, using Mini Transfer Packs or Midi Transfer Packs, with Trans-Blot® Turbo TM (Bio-Rad, Segrate, Italy) according to manufacturer’s instructions (Bio-Rad, Segrate, Italy).

The membrane was blocked in 5% skim milk (Cat. 70166, Sigma, Milan, Italy) for 45’ at room temperature. Subsequently membrane was incubated with indicated primary antibody, overnight at 4 °C in agitation. Primary antibodies used were: anti-GLAST (rabbit, 1:500, Cat. NB100-1869, Novusbio, Abingdon, UK), anti-puromycin (1:1000, Cat. MABE343, Millipore, Darmstadt, Germany), anti- β-Actin (mouse, 1:800, Sigma, Cat. A1978, Milan, Italy) was used to normalize protein load. Goat anti-mouse IgG (H + L) horseradisch peroxidase-conjugated secondary antibody (1:5000; Cat. 170-6516, Bio-Rad, Segrate, Italy) and Goat anti-mouse Igg (H+L) horseradish peroxidase-conjugated secondary antibody (1:5000; Cat. 170-6515, Bio-Rad, Segrate, Italy). Detection was carried out with SuperSignalTM West Pico PLUS Chemiluminescent Sbustrate (Thermo Scientific, Milan, Italy), based on the chemiluminescence of luminol and developed using ChemiDocTM Imaging System (Bio-Rad, Segrate, Italy). Quantitative densitometry of protein bands analysis was performed with ImageLab software. 

### 4.9. RNA Extraction and Real-Time PCR

Total mRNA was extracted from 1.0 × 10^6^ cells using TRIzol Lysis Reagent (Cat. 15596026, Invitrogen, Milan, Italy) according to manufacturer’s instruction. First strand of cDNA was synthesized from 0.5–1 µg of total RNA using SensiFast kit (BioLine, London, UK, Cat. BIO-65054). Real-Time PCR was performed using iTaq qPCR master mix according to manufacturer’s instructions (Cat. 1725124, Bio-Rad, Segrate, Italy) on a SFX96 Real-time system (Bio-Rad, Segrate Italy). To normalize raw real time PCR data, S18 ribosomal subunit was used. Primer sequences are provided in Table 1. Data are expressed as delta-C(t) of gene of interest to S18 allowing appreciation of single gene expression level. 

### 4.10. Statistical Analysis

Statistical analysis and related graphical representations was done using GraphPad Prism v.7. A two-tailed unpaired Student’s t-test or one way ANOVA test ware used. Differences were considered significant at *p* < 0.05.

## Figures and Tables

**Table 1:** List of oligonucleotide primers used for real-time PCR.

Protein Name	Gene Name	Forward/Reverse	Accession No.
S18	Rps18	TGCGAGTACTCAACACCAACA	NM_011296
mouse and human		CTGCTTTCCTCAACACCACA	NM_022551.3
EAAT1/GLAST mouse	Slc1a3	AATGCCTTCGTTCTGCTCAC	NM_148938
		ATCCTCATGAGAAGCTCCCC	
EAAT1/GLAST human	Slc1a3	GTTGCTCCGTTGTACCTGCT	NM_004172.4
		GGAATCACCCACAGAAAGCC	
